# Summary of the BSR Annual Meeting 2017

**DOI:** 10.5334/jbr-btr.1442

**Published:** 2017-11-18

**Authors:** Raymond Oyen

**Affiliations:** 1 UZ Leuven, BE

Dear colleagues,

The third Annual Meeting new style of the BSR is the result of a joint effort of the Section of Neuroradiology and the Section of Pediatric Radiology, with the highly appreciated input by the Young Radiologist Section (YRS).

An attractive and challenging program by a national and international faculty of renowned experts is offered to general radiologists and trainees. Neuroradiology has always been largely attractive, with continuously evolving knowledge and imaging techniques, and additional processing and post-processing software to refine diagnosis and – ultimately – therapy. Pediatric radiology deserves equal attention; indeed, especially for the pediatric population, dedicated imaging modalities have to be carefully used to guarantee safe conditions and accurate diagnosis in all circumstances.

The European Society of Radiology, the Radiological Society of North America and the American College of Radiology launched the International Day of Radiology (IDoR) a few years ago. The main theme for 2017 is on Emergency Radiology, a theme almost all radiologists are faced with in daily practice.

It is a great pleasure to welcome you at the 2017 Annual Meeting of the BSR in Brussels. Enjoy meeting science, colleagues and industry.

We are already looking forward to the 2018 Annual Meeting of the BSR; the main topics will be Head & Neck Radiology and Interventional Radiology.

Best regards.

Prof. Dr. Raymond Oyen

On behalf of the Scientific Council of the BSR

November 2017

